# A Pore-Facing Glycan Determines GABAA Receptor Subunit Stoichiometry and Gating Behavior

**DOI:** 10.21203/rs.3.rs-7743743/v1

**Published:** 2025-10-17

**Authors:** Jing Li, Amin Akbari Ahangar

**Affiliations:** University of Mississippi; University of Mississippi

**Keywords:** GABAA receptor, pentameric ligand-gated ion channel, N-glycosylation, hetero oligomeric complexes, molecular dynamics simulation, neurotransmitter

## Abstract

The assembly and gating of γ-aminobutyric acid type A receptors (GABA_A_Rs) are tightly regulated by their hetero-pentameric subunit composition, yet the molecular determinants governing the pentameric form remain elusive. Here, we demonstrate that a conserved *N*-linked glycan on α subunits, uniquely positioned within the central pore of the extracellular domain, acts as a structural gatekeeper limiting α subunit incorporation. Using a total of 28 μs of molecular dynamics simulations across native and putative GABA_A_Rs assemblies, we show that introducing a third pore-facing glycan or positioning two glycans on adjacent subunits disrupts key interfacial salt bridges and hydrogen bonds, particularly at the β+/α− interface that hosts the GABA binding site. These disruptions propagate allosterically, reduce internal loop flexibility, and alter extracellular-to-transmembrane domain coupling, ultimately leading to deep closure of the activation and desensitization gates in the transmembrane domain. Systems containing three glycans consistently shift toward dehydrated, non-conductive conformations. In contrast, native form with two pore-facing glycans preserved native interfacial networks and pore radius. Our findings provide a mechanistic insight for the long-observed α-limiting assembly pattern and identify glycan-mediated steric hindrance as a critical factor of receptor gating. These insights bridge evolutionary conservation, *N*-glycosylation, and dynamic structure-function relationships, highlighting pore-facing glycosylation as a key determinant of GABA_A_Rs architecture and function.

## INTRODUCTION

γ-Aminobutyric acid type A receptors (GABA_A_Rs) are integral to the central nervous system (CNS), functioning as chloride ion-selective channels that mediate inhibitory neurotransmission^1–5^. These GABA_A_Rs play critical roles in modulating neuronal inhibition, synaptic plasticity, and homeostatic control^6,7^. GABA_A_Rs dysfunction has been implicated in a wide range of neurological and psychiatric disorders, including epilepsy, schizophrenia, anxiety disorders, depression, and substance abuse^4,8–14^.

As pentameric ligand-gated ion channels (pLGICs), GABA_A_Rs assemble into pentamers from a diverse pool of 19 subunits (α1–6, β1–3, γ1–3, ρ1–3, δ, ε, π, and θ)^4,15,16^. Each subunit comprises a bulky extracellular domain (ECD) that forms the orthosteric binding sites for GABA and other ligands and four transmembrane helices that constitute the transmembrane domain (TMD), wherein the activation gate and desensitization gates of the channel are located^17–20^. Although the extensive repertoire of subunits could lead to a theoretical array of ~ 490,000 possible pentameric combinations, only a limited number of assemblies are observed *in vivo*^21*,*22^. Among the possible compositions, the αβγ is identified as the most predominant GABA_A_Rs assembly in the brain^21,23^. For instance, over 60% of synaptic GABA_A_Rs are composed of the α1β2γ2 subunit composition^6,18,21,23,24^ while the α2β3γ2 isoform accounts approximately 13% of the total GABA_A_Rs population^11^. Overall, the β-α-β-α-γ is believed to be the most abundant form of this channel^25,26^.

The physiology and pharmacology of GABA_A_Rs are shaped by their pentameric subunit composition^2–4,6,22,25,27^, which dictates receptor trafficking, ligand binding, drug responses, gating properties, and distinct forms of neuronal inhibition or cellular signalling^3,9,22,25,28,29^. Different subunit combinations target receptors to specific subcellular compartments. For example, γ2-containing receptors predominantly localize to synapses, whereas δ-containing receptors are primarily extrasynaptic, mediating phasic and tonic inhibition respectively^24^. Different assemblies also form unique subunit interfaces, enabling diverse ligand/drug binding and functional responses^22^. For instance, the GABA-binding site is located at the β+/α− interface, while the histamine-binding site is situated at the β+/β− interface^22^. Another example of the significance of subunit composition is that γ2 containing GABA_A_Rs (e.g., α1β2γ2, α2β3γ2, α3β3γ2) are benzodiazepine-sensitive, while δ-containing receptors (e.g., α4β2δ, α6β3δ) are benzodiazepine-insensitive but highly responsive to neurosteroids like allopregnanolone^25,30^.

The assembly rules and molecular determinants governing native GABA_A_Rs remain largely unknown, representing a fundamental challenge in understanding the biophysics and pharmacology of the GABA_A_ receptors. Extensive research has been conducted to elucidate the subunit composition of GABA receptors using biochemical, electrophysiological, and structural techniques. Experiments using concatenated constructs have revealed certain assembly patterns governing receptor stoichiometry, localization, subunit-specific preferential interactions, and probable/non-probable structure compositions^26,31–33^. High-resolution cryo-EM studies have uncovered representative subunit assemblies, revealing structural differences that shape receptor function and drug-binding properties^3,22,25,34–36^. These studies have identified a number of distinct native GABA_A_R pentamers, including canonical and non-canonical subunit assemblies. Among all available GABA_A_R structures, most of these subunit assemblies follow an XαXαX assembly pattern, with no native pentamers containing more than two α subunits or two adjacent α subunits. However, the underlying mechanisms behind this puzzling phenomenon remain unclear.

*N*-linked glycosylation of GABA_A_R s has been hypothesized to affect the XαXαX assembly pattern^37^, but it has not yet been investigated as a potential molecular determinant for receptor assembly. Among post-translational modifications, *N*-linked glycosylation is a critical regulator of protein folding, trafficking, and function in ligand-gated ion channels^38,39^. In GABA_A_Rs, glycosylation can be seen either as pore-facing glycans or surface glycans, influencing receptor biogenesis, gating, and assembly^40–42^. Glycosylation of GABA_A_Rs are mostly emphasized for their impact on potential subunit interactions and shielding of ligand-binding sites^37,40,43^, while fewer studies have investigated whether glycosylation alters receptor subunit assembly pattern. Cryo-EM studies have characterized these important glycan structures, including a Man-8 structure at N123 in the extracellular domain (ECD) of the α subunits, positioned centrally within the receptor’s pore^22,34,37,44,45^. This pore-facing glycan is of particular interest owing to its spatial location, which is hypothesized to impact pentameric composition, structural stability, or ion permeability^37,45,46^. Interestingly, any resolved structure that involves an α homomeric structure has been subjected to extensive modifications that would disrupt the natural pore-glycosylation on α subunits^47,48^. Despite the glycan’s critical location, the molecular mechanisms by which pore-facing glycans affect channel composition and gating remain largely unexplored.

We hypothesize that the presence of pore-facing glycans at α1N123 likely prevents the formation of pentamers with more than two α subunits or with two adjacent α subunits, due to steric clashes. This steric constraint may promote the incorporation of β and γ subunits, thereby favoring the canonical XαXαX assembly pattern. In this context, this pore-facing glycan may serve as a molecular determinant guiding subunit composition and spatial arrangement for receptor assembly. To test our hypothesis, we investigate the structural and functional implications of high-mannose glycosylation at N123 of the α1 subunit using molecular modeling and molecular dynamics (MD) simulations. Different glycosylated and non-glycosylated systems and putative assemblies of the channel were investigated. Based on the detailed analysis of MD trajectories, we characterized the impact of these structural variations on the subunit interface, their allosteric effects on TMDs, and the coupling mechanisms underlying signal propagation.

## MATERIAL AND METHODS

### Molecular Dynamics Simulation

#### System setup:

**Figure 1:**
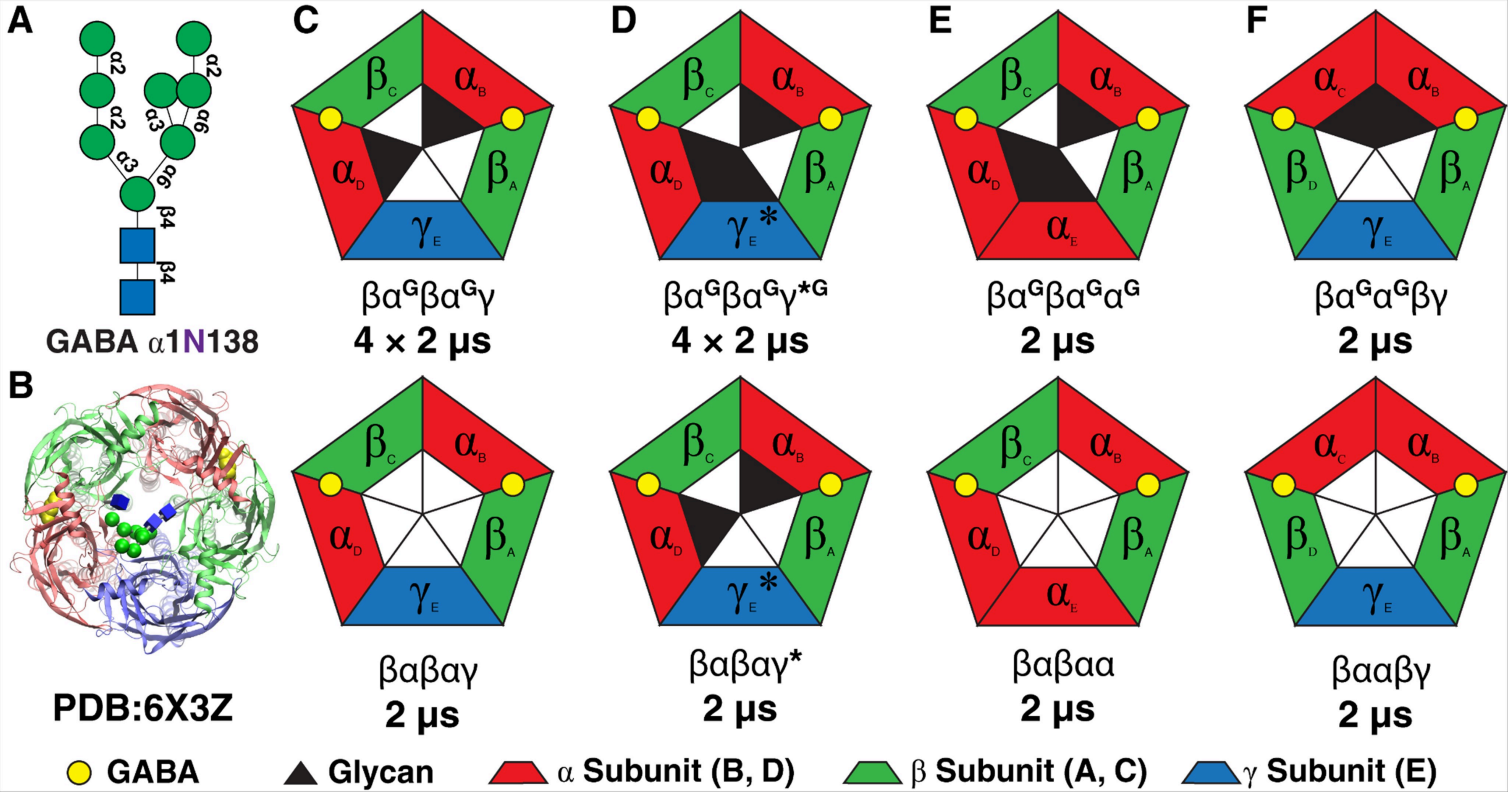
Proposed systems for investigating the effect of the pore-facing *N*-glycosylation on GABA_A_R Gating and their controls with no pore-facing glycans. A) The high mannose structure of the pore-facing glycan. B) Top view of the PDB structure used for modeling the systems with man-8 structure in the middle, C) wild-type system with two pore-facing glycans (βα^G^βα^G^γ) and the same pentameric form without pore-facing glycans (βαβαγ), D) The mutant (W162N in γ subunit) with three pore-facing glycans (βα^G^βα^G^γ*^G^) and the glycan-free equivalent form (βα^G^βα^G^γ*). E) system with three α subunits and three pore-facing glycans (βα^G^βα^G^α^G^) and the glycan-free form (βαβαα). F) system with two α subunits adjacent to each other and two pore-facing glycans (βα^G^α^G^βγ) and the glycan-free form (βααβγ).

All atomic models of GABA_A_R were constructed (Fig. 1) based on the wild-type (WT) cryo-EM structure (Fig. 1A)^44^ (PDB ID: 6X3Z) with and without the pore-facing glycans. Putative assemblies of the channel were constructed by structural alignment of appropriate subunits on the cryo-EM structure using VMD’s^49,50^ MultiSeq^51^. For all the MD simulations, the channel comprising different assemblies was embedded in a 115×115 Å bilayer composed of POPC lipids after the assignment of its orientation through the PPM 2.0 server^52^ and solvated in 150 mM NaCl using the web service CHARMM-GUI^53,54^. Most residues were assigned their default protonation state at pH 7.0 based on the PROPKA3^55^ prediction obtained. The GABA molecule was protonated using CHARMM Ligand Reader & Modeler^56^. The man8 structures were constructed and attached to both α1N138 residues in CHARMM-GUI Glycan Reader & Modeler^57–59^. Disulfide bonds were introduced at α_B, D_ 166–180, β_A, C_ 160 – 74, and γ_E_ 190–204 to conserve the integral Cys-loop structure. The total number of atoms in each system was approximately 165,000.

### Simulation protocol

The CHARMM36m force field for protein^60–62^, lipids^63^, ions^64^, and glycans ^65^ were used. Explicit water was described with the TIP3P model^66^. All the simulations were performed under constant number of particles N, pressure P, and temperature T (NPT) conditions using the Nosé–Hoover Langevin piston method to maintain the pressure at 1 atm and a Langevin thermostat to maintain the temperature at 310 K^67^. The oscillation period of the piston was set at 100 fs and the damping time scale at 50 fs. Long-range electrostatic interactions were calculated using the particle mesh Ewald algorithm^68^ with a grid spacing of 1 Å. All simulations were performed under tetragonal periodic boundary conditions to the simulation box to overcome finite-size effects and mimic bulk-like properties. Long-range electrostatic interactions were calculated using the particle mesh Ewald algorithm^68^. Short-range nonbonded interactions were calculated with a cutoff of 12 Å, and the application of a smoothing decay started to take effect at 10 Å. The simulations used the SHAKE algorithm^69^ to fix bond distances involving hydrogen atoms and applied hydrogen mass repartitioning^70^ to reweight hydrogen atoms, allowing for a 4 fs time step for MD simulations. After 5000 steps of minimization and equilibrations for 2 ns with harmonic positional restraints (k = 1 kcal/mol/Å^2^), each equilibrated system was simulated for at least 2 μs using NAMD2.14^71^ and NAMD3^72^ on expanse or Anton3 resulting in a total simulation time of 28 μs. Trajectories were then analyzed using VMD^50^ and Python scripts.

Production simulations on Anton3^73^ were performed under the NPT conditions using Desmond^74^ with the Multigrator integrator^75^. Semi-isotropic pressure control was applied using a Monte Carlo barostat and the antithetic thermostat^75,76^ to maintain a pressure of 1.0 atm and a temperature of 310 K. A 2.5 fs time step was used with RESPA^77^ for multiple time-scale integration. Electrostatic interactions were computed using the u-series method and Midtown splines^78,79^. Nonbonded interactions were tapered using a force-shift scheme with a 12 Å cutoff.

### Analysis and Visualization

#### Salt Bridge Network Analysis

To assess the salt bridge network at the subunit interfaces, we monitored the distance between oppositely charged residues over time throughout the simulations. A salt bridge was defined as the presence of the terminal carbon atom of glutamate or aspartate side chain within 4 Å of either the terminal carbon of an arginine or the terminal nitrogen of a lysine, within a given frame. The analysis was conducted across all independent simulation runs for the same construct. The distances were standardized and averaged, and standard errors were calculated. Salt-bridge pairs were included in the comparison between systems only if the occupancy difference exceeded 10%. This strategy enabled a detailed and comprehensive assessment of inter-subunit salt bridge networks.

#### Hydrogen Bond Analysis

The hydrogen bonds at the interface were analyzed using the Hydrogen Bond module in VMD. Polar atoms (N, O, S, F) were evaluated with a distance cutoff of 3.5 Å and a donor-hydrogen-acceptor angle threshold of 20°. The occupancy of each hydrogen bond pair was calculated as the percentage of frames in which the bond was present across the simulation trajectories, averaged over all frames, and the standard error was computed on the basis of multiple independent runs. Only hydrogen bond pairs with an occupancy difference greater than 10% between systems were included in the final comparison.

#### ECD expansion analysis

Expansion of the protein’s ECD was assessed by tracking the 2D (on XY plane) center of mass (COM) of the ECD for each subunit throughout each simulation trajectory using MDAnalysis^80^. For each frame, the area enclosed by these COM points was determined using the ConvexHull function in python library SciPy^81^. The resulting area values were averaged across simulation replicas and compared between different conditions to evaluate differences in ECD expansion.

##### Internal Loop RMSD Analysis:

The flexibility of internal loops was assessed by calculating the root-mean-square deviation (RMSD) of the backbone atoms for the equivalent loop in each subunit (α: residue 130–143, β: 124–137, γ: 154–167) for every frame, using the first frame as the reference. The RMSD values were averaged across trajectories and compared between different conditions to evaluate the flexibility of the internal loop.

#### Glycan distribution analysis

The distribution and orientation of glycans were measured by tracking coordinates of the Cα of each glycosylated asparagine (αN138, γN162) and the terminal mannose residue of each glycan for every frame. This approach allowed for tracking glycan movement and orientation throughout the simulations.

##### Pore radius analysis:

The pore diameter along the z-axis (vector [0,0,1]) was analyzed using the HOLE2 module^82^ of the MDAnalysis package (version 2.6.1)^83^ to quantify changes in pore radius. The central reference point in each simulation was determined as the collective center of mass (COM) of Cα residues forming the activation gate. The end radius for the HOLE analysis was set to 25 Å, and the pore radius at regions of interest was tracked throughout each simulation replica. Three distinct gate regions were specifically analyzed: 1) the activation gate (AG) located at position 9′^84^, the second hydrophobic constriction site (2nd HCS), corresponding to residue α1V257, and the homologous position in other subunits, crucial for maintaining the structural integrity of the pore lining, proper packing of the channel-forming helices, and overall channel function^85,86^, and the desensitization gate (DG) at −2′^84^.

#### Water occupancy analysis

Water occupancy was assessed by tracking water molecules within a 5×5 Å^2^ area along the z-axis at the central pore. The position of each water molecule was determined based on the coordinates of its oxygen atom. Water distribution along the channel pore was quantified by counting water molecules within 2 Å bins along the z-axis throughout the simulation trajectory. These values were averaged across independent simulation runs. To evaluate water permeability as a proxy for channel functionality, we used a representative water molecule radius of 1.25Å^87^ for cutoff, classifying regions as permeable (radius > 1.25 Å) or non-permeable (radius ≤ 1.25 Å). The number of permeable versus non-permeable pore in each region was recorded accordingly. Statistical significance was evaluated using the chi-squared test, complemented by a sensitivity test to assess robustness, using Scipy and statsmodels.

#### Allosteric coupling analysis

To characterize the allosteric coupling between the ECD and transmembrane domain (TMD), we performed dynamical network analysis using simulation trajectories. The analysis involved multiple steps, including contact map generation, network construction, and pathway extraction to identify the coupling pathway and key residues mediating structural communication within the protein. Network configuration included the protein and glycan, with hydrogen atoms excluded. Network nodes were defined as backbone Cα atoms for the protein and O5 atoms for the glycans. To avoid artificially short paths, edges between nodes representing neighboring residues (e.g., residue *i* and *i* + 1) were excluded to eliminate trivial correlations arising from backbone adjacency, allowing the analysis to focus on long-range communication within the system. Network analysis was executed using VMD’s NetworkView package^88^, which processed contact maps from each independent run. The shortest paths between key functional residues were extracted using suboptimal path algorithm^88^. The algorithm computed frequent residue paths, identifying highly connected residues that serve as communication hubs. The betweenness centrality of each node was computed to rank residues based on their importance in information transfer. The most frequently visited residues in each path were extracted. Residues appearing in multiple independent runs were considered conserved network hubs contributing to allosteric signaling. VMD, along with Python^89^ packages Numpy^90^, Pandas^91^, and Matplotlib^92^, were used to visualize the data from all of the analyses.

## RESULTS

### Evolutionary Conservation and Specificity of the Pore-Facing Glycan in the α Subunits of GABA_A_Rs

**Figure 2:**
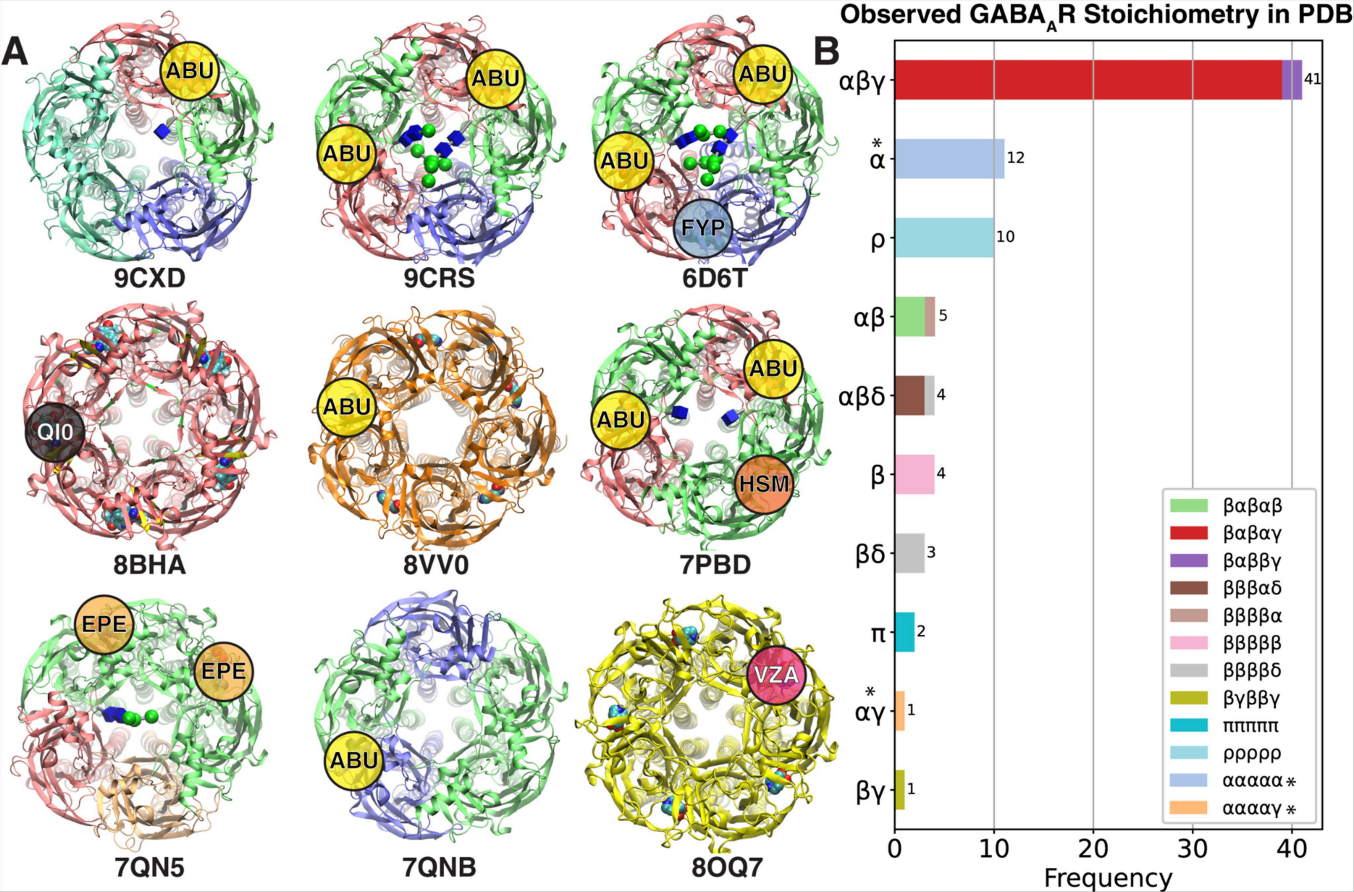
Structural survey of the available 3D structures of GABA_A_Rs. A) Examples of previously resolved GABA_A_R structures, color coded based on subunits (Green: β, Blue: γ, Red: α, Orange: π, Yellow: ρ, Orange: δ) in complex with different small molecules (ABU: GABA, FYP: Flumazenil, QI0: Basmisanil, HSM: Histamine, EPE: HEPES, VZA: TPMPA) B) A survey of composition frequency of GABA_A_R’s resolved structures. *The pore-facing glycosylation sites are mutated in these structures.

A survey of 85 available 3D structures of GABA_A_R in the PDB database (till 2025–02-22) shows the dominance of the αβγ stoichiometry (Fig. 2). The majority of the entries in this category take a βαβαγ configuration^22,34,36,44,45,93–97^, with recent structures highlighting the possibility of incorporating different α subunits within the same assembly^34^. Interestingly, a few structures are reported with more than two α subunits (Fig. 2A). However, such structures undergo substantial modifications at the extracellular domain that remove native N-glycosylation sites, resulting in ECDs that differ significantly from natural α subunits. Notably, in all available structures with a native α subunits, the first few N-acetylgalactosamine (GalNAc) residues of the high mannose glycan are observed, whereas these glycans are entirely absent in the ECD-modified α homomeric assemblies or ααααγ forms. This points toward the possibility for the pore-facing glycans to hold the key to GABA_A_R assembly pattern.

**Figure 3:**
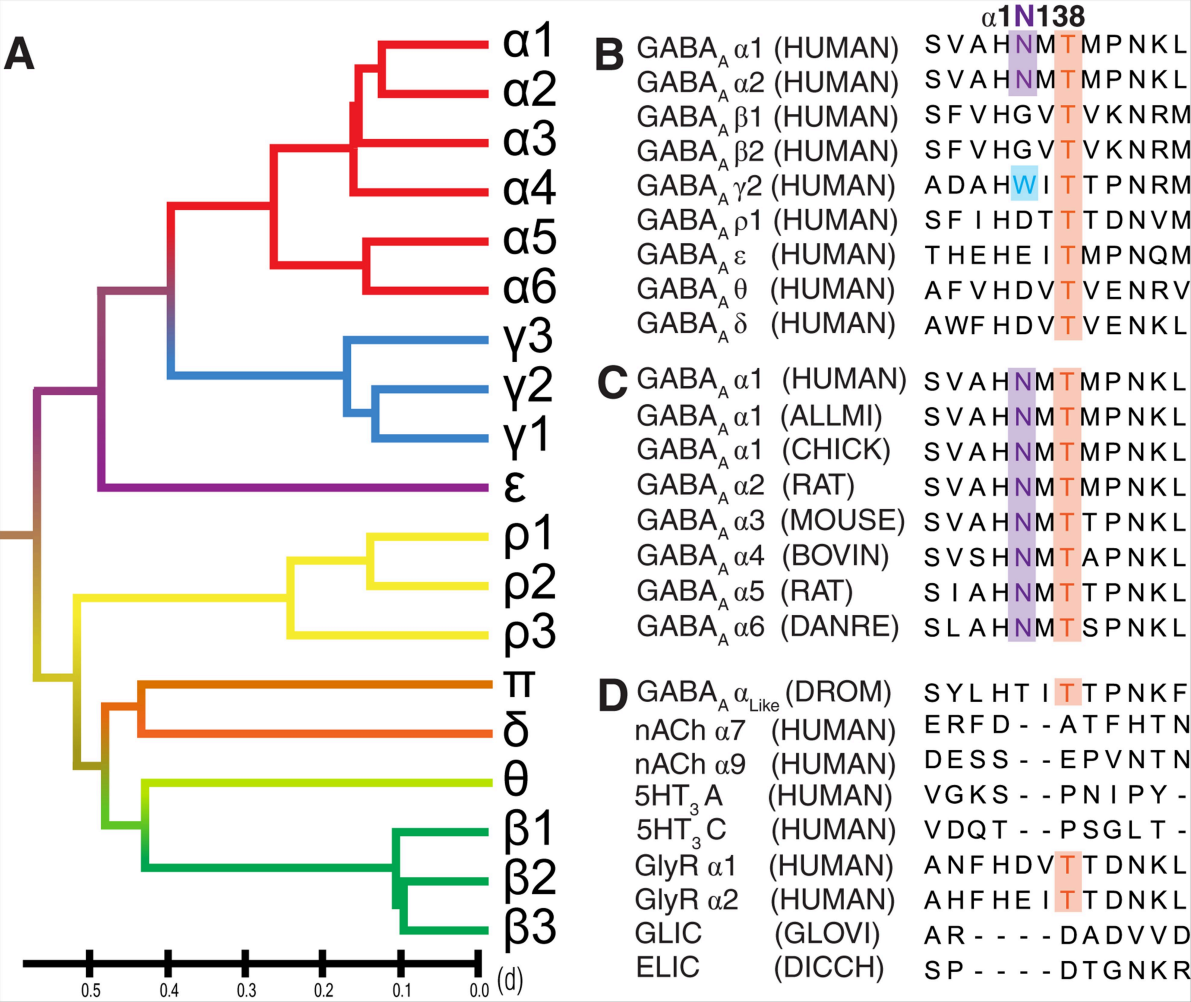
Sequence analysis of the GABA_A_Rs. A) Phylogenetic tree of GABA_A_Rs subunits, highlighting the close evolutionary relationship between γ and α subunits. B) Multiple sequence alignment (MSA) of different subunits within human GABA_A_Rs. C) MSA of α subunits across different species. D) MSA of the different members of the pLGIC family that can form homomers.

Multiple sequence alignment of human GABA_A_R subunits revealed that the pore-facing *N*-glycosylation site at α1N138, is highly conserved across all α subunits (Fig. 3). Positioned centrally within the ECD pore, this hallmark glycosylation site is uniquely observed in the α subunits of GABA_A_R (Fig. 3). This glycosylation sequon lies within the loop A-E linker region. Both the sequon and the loop itself show high evolutionarily conservation among different α subunits across various species (Fig. 3C). Interestingly, the pore-facing glycan appears unique to vertebrates, including birds, fish, reptiles, and mammals, whereas more evolutionarily distant GABA_A_R α_like_ subunits found in fruit flies lack the asparagine residue necessary for *N*-glycosylation (Fig. 3D). Notably, this pore-facing glycosylation site is absent in any other GABA_A_R subunits (Fig. 3B) and any other known member of the pLGIC superfamily (Fig. 3D). Sequence analysis also identified a tryptophan residue (γ2W162) occupying the homologous glycosylation site in the γ2 subunit (Fig. 3B). Phylogenetic analysis indicates closer evolutionary relatedness between γ and α subunits than other GABA_A_R subunit classes (Fig. 3A), suggesting γ2W162 as a promising target for engineering a comparable pore-facing glycosylation site. Mutating γ2W162 to asparagine would introduce the sequon for *N*-glycosylation to attach a third glycan in the central pore without significantly altering the native channel structure.

### The native, mutated, and putative models to test the structural roles of pore-facing glycans

Atomic models of GABA_A_R were constructed (Fig. 1) based on the WT cryo-EM structure (Fig. 1A)^44^ (PDB ID: 6X3Z) with and without the glycans (βα^G^βα^G^γ, βαβαγ). Mutation W162N was introduced at the homologous position of α1N123 on the γ2 subunit of the βα^G^βα^G^γ*^G^ system (Fig. 1D), to explore the channel’s ability to accommodate a third glycan without significantly disrupting the native structure. Putative assemblies with three α subunits and three pore-facing glycans (βα^G^βα^G^α^G^, Fig. 1E), or with two adjacent α subunits bearing glycans (βα^G^α^G^βγ,Fig. 1F), were also constructed to probe their structural stability. Non-glycosylated counterparts (βαβαα and βααβγ) served as controls. All these putative assembly models were generated by structural alignment of equivalent subunits to the cryo-EM structure using VMD’s MultiSeq^51^. Each assembly was simulated for at least 2 μs to investigate the structural impacts of the pore-facing glycans across different pentameric configurations.

### The additional pore-facing glycan disrupts key native inter-subunit contacts in the ECD of βα^G^βα^G^γ*^G^

**Figure 4:**
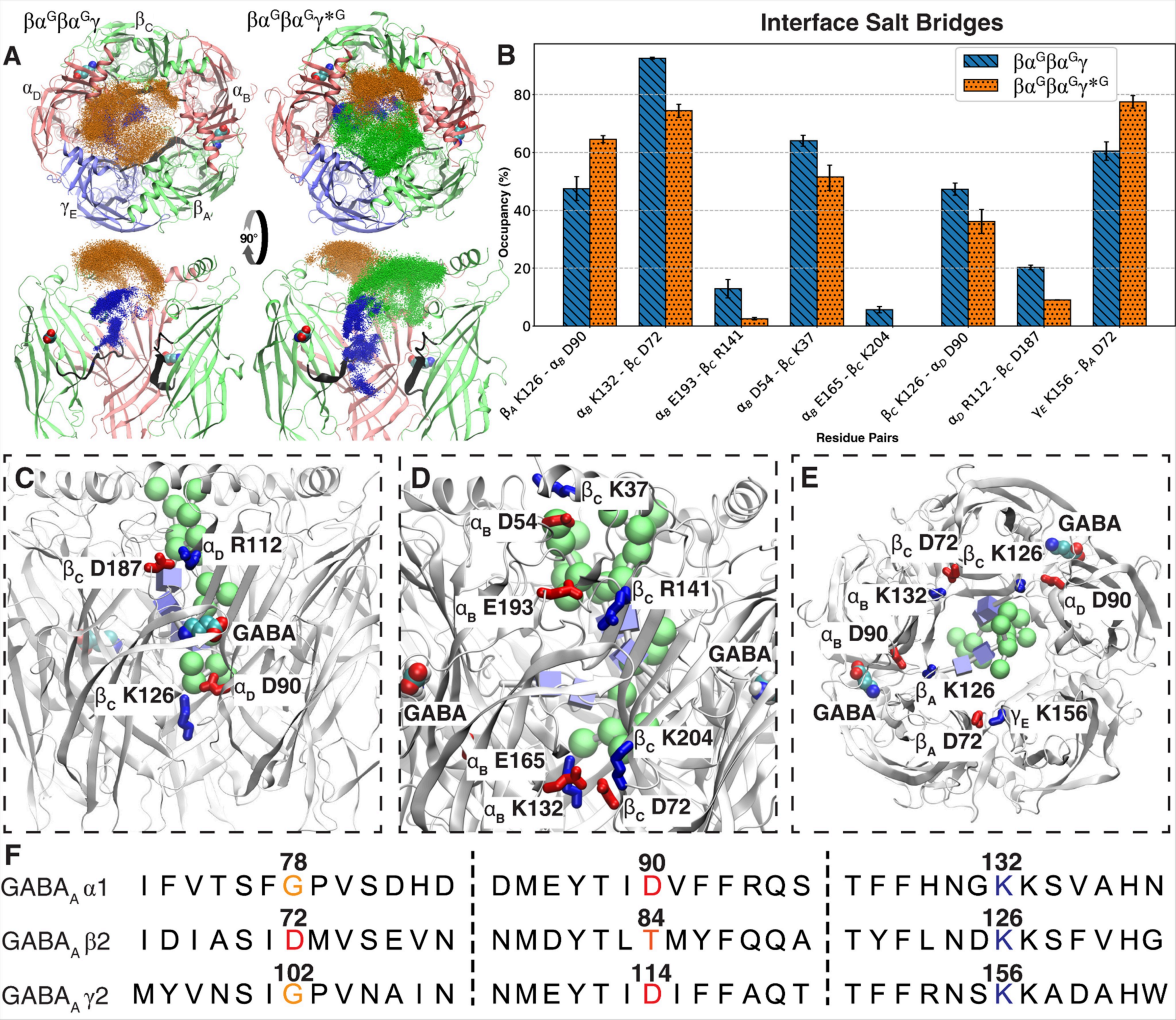
The effect of additional glycosylation in βα^G^βα^G^γ*^G^ on salt bridges at ECD subunit interface. A) Distribution of terminal mannoses in βα^G^βα^G^γ*^G^ and βα^G^βα^G^γ (Blue: CARA attached to α_B_, Orange: CARB attached to α_D_, Green: CARC attached to γ_E_). B) The subunit interface salt bridges significantly altered between βα^G^βα^G^γ*^G^ and βα^G^βα^G^γ. C) Disrupted salt bridges at GABA-binding site located at β_C_ - α_D_ interface. D) The disrupted salt bridge at the α+/β− interface. E) The altered salt bridges by the additional glycan at similar locations in the lower parts of ECD. F) Multiple sequence alignment shows several affected salt bridges are located at equivalent positions at the lower ECD.

The incorporation of the third glycan at γ_E_ ECD in the engineered central pore in βα^G^βα^G^γ*^G^ leads to altered glycan localization and the disruption of native inter-subunit salt bridges and hydrogen bonds. Both βα^G^βα^G^γ*^G^ and βα^G^βα^G^γ were simulated in four independent 2-μs runs (Fig. 1C&D). Comparison of these trajectories revealed reproducible patterns in the structural impacts of the three pore-facing glycans. Firstly, this additional glycan significantly (*p* = 0.036) reduces the conformational flexibility of the glycan attached to α_D_ in the βα^G^βα^G^γ*^G^ (RMSF:4.14 ± 0.45) compared to the βα^G^βα^G^γ (RMSF:6.59 ± 0.93) system (SFig. 1). The flexibility of α_B_ glycan is also reduced, but this effect is not statistically significant (*p* = 0.22). The visualization of the terminal glycans shows that the additional γ_E_-attached glycan forces the α_D_-attached glycan to be concentrated at the α_B_ - β_C_ and β_C_ - α_D_ interface (Fig. 4A), sterically repulsing those subunits away from each other.

This structural rearrangement changes the native contacts at the subunit interfaces, resulting in a substantial reduction (> 10%) in native salt-bridge occupancy for most affected pairs (Fig. 4B). This reduced salt bridge occupancy is particularly pronounced at the GABA binding site located at the β_C_ - α_D_ interface (Fig. 4C), whereas the β_A_ - α_B_ binding site is less affected (Fig. 4B). This result aligns with the observation that the α_D_-attached glycan repulse the α_B_ - β_C_ and β_C_ - α_D_ interface. Notably, several disrupted interactions, such as β_A_ K126 - α_B_ D90, α_B_ K132 - β_c_ D72, β_c_ K126 - α_D_ D90, and γ_E_ K156-β_A_ D72, are altered at nearly symmetrical locations across the subunit interfaces (Fig. 4E). In each of these pairs, the lysine residues occupy homologous positions (Fig. 4F), whereas the aspartates are situated in similar locations within adjacent β sheets (Fig. 3F, Supplementary Fig. 1), in proximity to different orthosteric binding sites. Among these, two pairs (β_c_ K126 - α_D_ D90 and α_B_ K132 - β_c_ D72) near the glycan-concentrated region exhibited reduced salt-bridge occupancy, whereas the other two pairs (γ_E_ K156 - β_A_ D72 and β_A_ K126 - α_B_ D90), located away from this region showed increased salt bridge occupancy (Fig. 4B, E). Although most disruptions occurred within the ECD subunit interface, several important changes in other key regions were observed, including α_B_ K306 - β_C_ E76 at the ECD-TMD interface (Supplementary Fig. 1) and α_D_ K339 - γ_E_ D299 within internal regions of TMD ( Supplementary Fig. 1).

**Figure 5:**
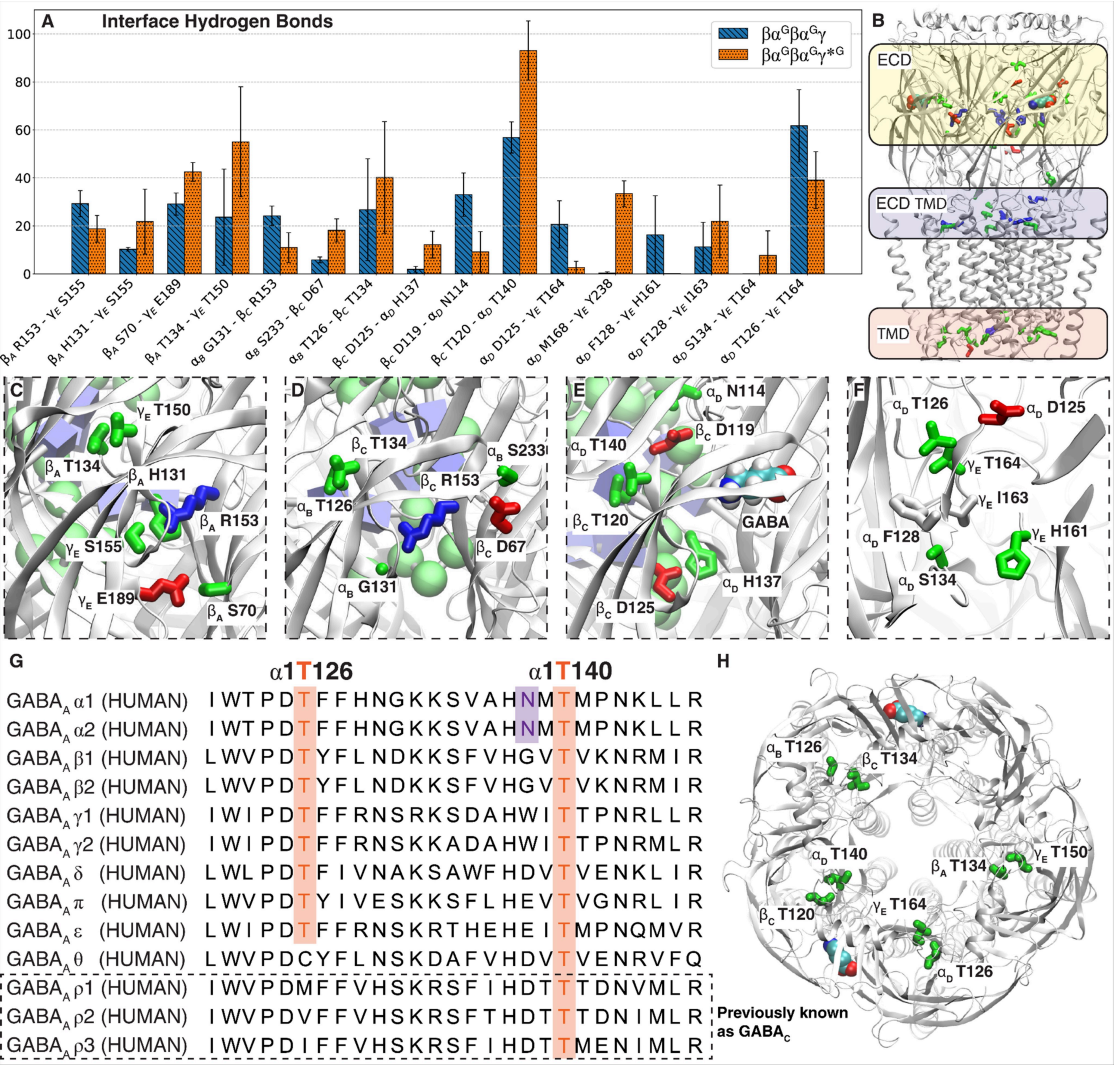
Rearranged hydrogen bond due to the introduction of the third glycan in βα^G^βα^G^γ*^G^. A) Hydrogen bond occupancy comparison between βα^G^βα^G^γ*^G^ and βα^G^βα^G^γ at ECD. B) The overall view of altered hydrogen bond clusters in the structure, C) Zoom in view of affected residue pairs at γ_E_-β_A_ interface, D) at α_B_-β_C_ interface, E) GABA binding site, and F) the benzodiazepine binding site. G) MSA of hGABA_A_R showcasing the conservation of the identified threonines involved in critical hydrogen bonds. H) Top view of the threonines with significant change in hydrogen bond occupancy.

Subunit rearrangements were also associated with altered hydrogen bonds at subunit interfaces which clustered into three primary regions, i.e., ECD, ECD-TMD interface, and TMD. (Fig. 5B, SFig. 2). At the ECD region, these re-arrangements were mostly observed around the discussed glycan-concentrated region, orthosteric and allosteric ligand-binding sites. At the β_C_–α_D_ interface, located behind the GABA binding site (Fig. 5A), several hydrogen bonds exhibited increased occupancy, including β_C_ D125–α_D_ H137 and β_C_ T120–α_D_ T140, whereas others, such as β_C_ D119–α_D_ N114, showed reduced occupancy. A similar pattern was observed at the α_D_–γ_E_ interface, which comprises the benzodiazepine binding site (Fig. 5A,E). In this region, hydrogen bonds such as α_D_ F128–γ_E_ H163 and α_D_ S134–γ_E_ T164 increased in frequency, whereas interactions including α_D_ D125–γ_E_ T164, α_D_ F128–γ_E_ H161, and α_D_ T126–γ_E_ T164 showed decreased occupancy. Overall, this analysis indicates that increased glycan-subunit interactions in βα^G^βα^G^γ*^G^ drive subunits separation at the upper surface of the ECD, while promoting closer contacts among residues in the lower, internal regions and loops.

Interestingly, at nearly all subunit interfaces except β_A_–α_B_, a hydrogen bond between two highly conserved threonine residues (Fig. 5G,H) was either significantly strengthened or significantly weakened (Fig. 5A). This threonine pair is located directly behind the orthosteric ligand-binding sites for GABA, benzodiazepine, and histamine. The second threonine in the α subunits contributes to the *N*-glycosylation sequon (Fig. 5G). Thus, alterations in this conserved hydrogen bond are likely affected by the presence of glycans and may have a direct structural impact on the ECD domain.

At the ECD–TMD interface, a second cluster of altered hydrogen bonds was identified, primarily involving the TM2–TM3 linkers and upper TMD regions (Supplementary Fig. 2). The most significantly affected interface in this region was γ_E_–β_A_, a potential binding site for steroid analogues and lipids (Supplementary Fig. 2). Structurally homologous positions at the propofol binding site in β_A_–α_B_, as well as β_C_–α_D_ interfaces, also exhibited disruption in their upper TMD regions. A third cluster of disrupted hydrogen bonds was located at the intracellular side of the TMD adjacent to TM1–TM2 linkers, regions known to bind neurosteroids, cholesterol, and other lipids (Supplementary Fig. 2).

The impact of the additional glycan extends beyond the disruption of native inter-subunit contacts. In the ECD, it also alters glycan interactions with important internal loops. Notably, the simulated pore-facing glycan of the γ subunit is positioned in close proximity to the functionally important A-E linker (Supplementary Fig. 1F), which contains the glycosylation sequon on the α subunit and is located behind the GABA binding site. RMSF analysis reveals a general decrease in loop flexibility, with the most pronounced reductions observed in the β_C_ and γ_E_ (SFig. 1F). This diminished internal loop flexibility provides an additional mechanism by which the third glycan may impair channel function.

### Presence of three pore-facing glycans in βαβαγ* lead to TMD closure at the activation gate

**Figure 6:**
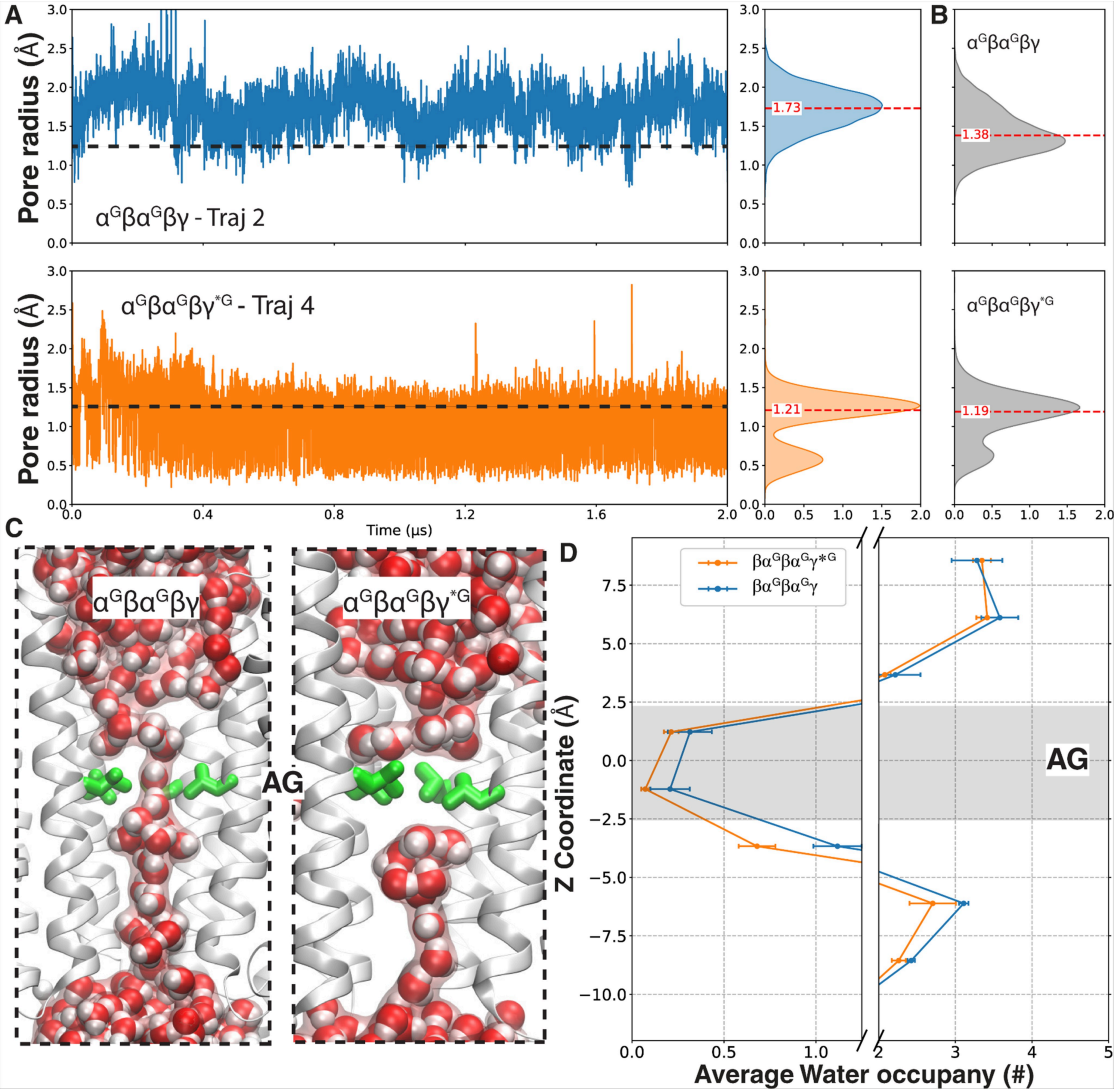
The effect of the third glycan in βα^G^βα^G^γ*^G^ on the TMD and gating dynamics. A) AG pore radius over time showing dynamic switching between closed and constricted states in the βα^G^βα^G^γ*^G^. B) Average AG radius across four independent simulations. C ) Representative water wire continuity at the activation gate in βα^G^βα^G^γ*^G^ versus βα^G^βα^G^γ. D) Water occupancy at the AG is lower across trajectories in βα^G^βα^G^γ*^G^.

The primary difference between the βα^G^βα^G^γ*^G^ and βα^G^βα^G^γ systems in the TMD are at the activation gate. Tracking the pore radius along the z-axis using HOLE analysis revealed a rapid closure of the AG where the pore radius is significantly (χ^2^ = 4717.44, *p* < 0.001) reduced from 1.38 Å in βα^G^βα^G^γ to 1.19 Å in βα^G^βα^G^γ*^G^ (Fig. 6A, B; SFig. 4). This increases the probability of channel closure (1.25 Å as the threshold) from 30.2% in βα^G^βα^G^γ to 59.8% in βα^G^βα^G^γ*^G^. Consistently, the βα^G^βα^G^γ system maintained a continuous water wire through the gate for nearly 43% of the simulation time, whereas in βα^G^βα^G^γ*^G^, this number dropped to 11% (Fig. 6C, D). This shift toward impermeable conformations likely results from a structural transition to a resting-like or deep desensitized conformation. The hallmark of this transition is the closure of the activation gate within the TMD, occurring at the crossover of key residues: α_B,D_ L291 β_A,C_ L283, and γ_E_ L313 (Fig. 6C). Interestingly, the βα^G^βα^G^γ*^G^ activation gate exhibited a distinct rapid-switching behavior, characterized by repeated transitions between a narrowed pore radius of ~ 1.25 Å and a fully sealed state (~ 0.6 Å) (Fig. 6A). This two-state-like switching behavior was consistently observed across all four βα^G^βα^G^γ*^G^ replicas. In contrast, the βα^G^βα^G^γ system predominantly maintained a pore radius above 1.25 Å. Furthermore, in a βα^G^βα^G^γ* with the same γ2W162 mutation without the glycosylation presence, the pore radius at AG remains similar (1.35 Å) to that of the βα^G^βα^G^γ system (1.38 Å) (SFig. 4), suggesting that the the observed dynamic behavior at this gate is driven by the glycan itself, rather than the mutation.

Eliminating all pore-facing glycans in the alternative pentameric configuration, βαβαγ, did not result in significant structural changes in either the ECD or the TMD, aside from modest rearrangements at the subunit interfaces (SFig. 3). The convex hull of the ECD remained stable, and the pore radius in this region was comparable to that of the βα^G^βα^G^γ. Changes in internal loop flexibility were minor and lacked a consistent pattern. Water-occupancy patterns of βαβαγ remained comparable to those observed in βα^G^βα^G^*γ* at the activation gate with only a subtle decrease at desensitization gate consistent with the latter’s minor decrease in pore radius at DG (SFig. 3, 4). Overall, in the absence of pore-facing glycans, the channel βαβαγ maintains structural and dynamic properties similar to those of βα^G^βα^G^*γ*, without substantial alterations in dynamic behavior.

### An Allosteric Network Coupling ECD Disruption to Activation Gate Closure

**Figure 7:**
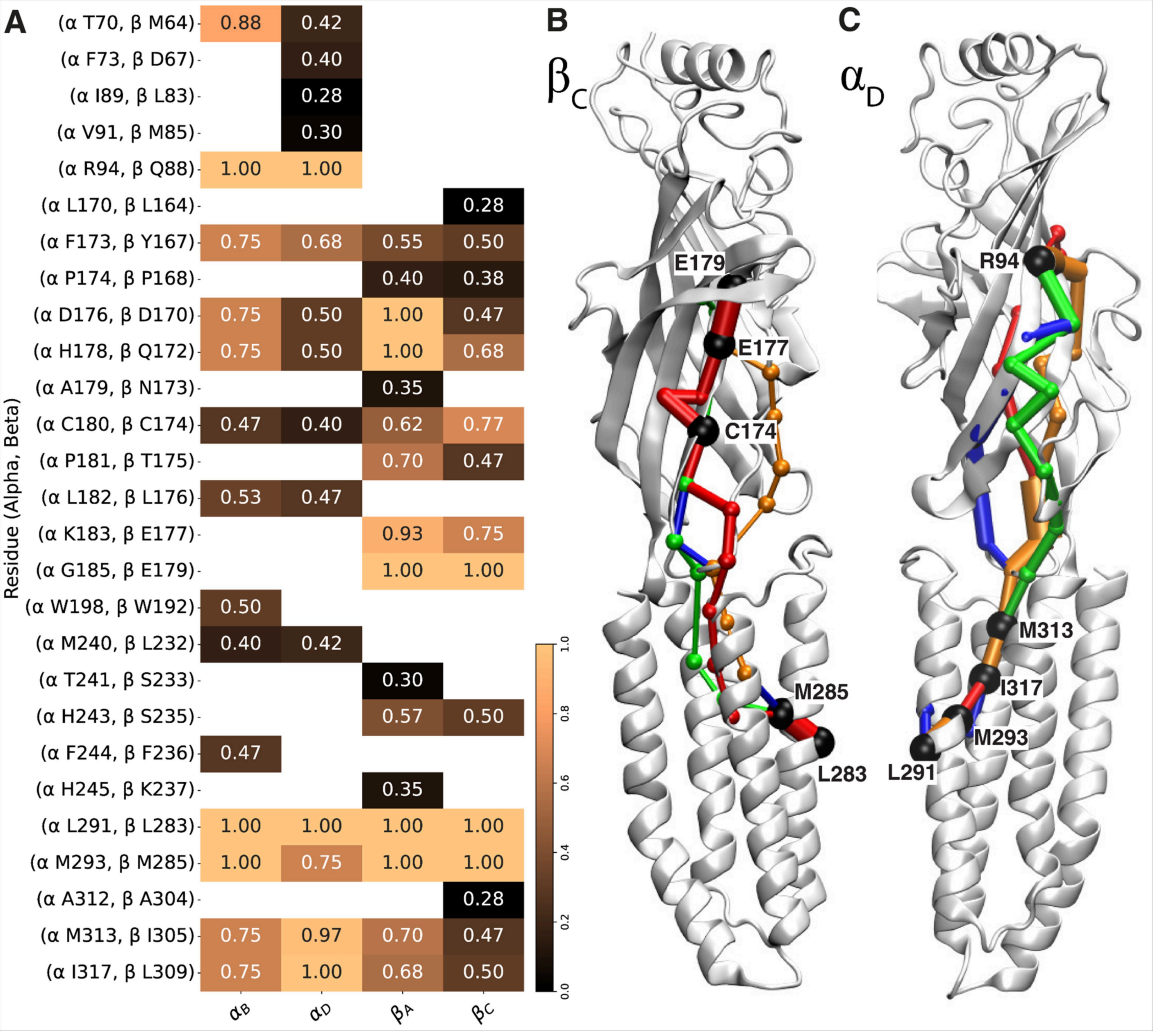
Allosteric coupled pathway connecting GABA binding site to AG. A) The pathways in each subunit and the corresponding recurrence in independent runs mapped on α - β MSA. pathway from each independent run (green, orange, blue, red) is visualized within B) β_C_ subunit and C) α_D_ subunit, with black spheres representing the residues with 75% recurrence. The thickness of the edges corresponds to the correlation value between the nodes.

The highly reproducible conformational changes observed in both the ECD and TMD across all βα^G^βα^G^γ*^G^ trajectories, particularly disruptions of native contacts in the ECD and activation-gate closure in the TMD, strongly suggest the presence of an allosteric coupling that transmits the structural effects of the additional glycan from the ECD to the TMD. Dynamical network analysis within each subunit revealed a consistent pathway originating from charged residues at the GABA binding site and extending to the activation-gate in the TMD (Fig. 7). These pathways commonly traverse the Cys-loop interface and the TM2-TM3 linker at the ECD-TMD junction, highlighting their role as key conduits for allosteric communication.

Within subunits approximately 93.9% of the observed residue-residue pairs exhibited correlation coefficients exceeding a threshold of |C_i_〿| ≥ 0.5, with a mean correlation of 0.718 (Supplementary Tables 3 and 4) indicating a strong and functionally meaningful coupling between structural elements^88^. This high correlation is strong evidence that the identified allosteric pathways are not artifacts of stochastic fluctuations, but instead reflect robust structural communication. These pathways consistently extend from the GABA binding site—located near the glycan attachment region—toward the activation gate, suggesting a directional and functionally relevant allosteric network. Notably, this network appears to be significantly modulated by glycan presence, reinforcing the role of glycosylation in shaping long-range signal propagation within the receptor.

Excluding the start and end residues, 12 out of 15 residues in the α subunit pathways appeared in both α_B_ and α_D_ subunits (Fig. 7A). Moreover, α_B_ shows higher occupancy conservation with 7 out of 12 residues appearing in more than 75% of trajectories, whereas α_D_ demonstrates greater pathway diversity and lower conservation at the ECD, with only 3 out of 13 residues conserved above the 75% threshold. This variability in α_D_ is attributed to its proximity to the glycan localization site, where glycan-induced displacement of subunits broadens the explored conformational space. In β subunits, 11 out of 16 residues were observed between both subunits across different pathways (Fig. 7). Within these subunits, β_A_ has 4 out of 14 residues with over 75% recurrence, whereas β_C_ has 3 out of 13 residues with repeated observation. Additionally, 7 residues (excluding AG residues) between α and homologous β subunit positions consistently appeared across all pathways, highlighting significant cross-subunit consistency in signal propagation (Fig. 7A). Overall, pathways demonstrate higher conservation within the TMD, while the ECD displays more diverse and dispersed pathway exploration.

Analysis of the protein–glycan interface edges reveal distinct interaction patterns among different glycans (SFig. 2). The α_D_-attached glycan (CARB), which exhibits the lowest fluctuations and strongest spatial localization at the β_C_-α_D_ interface, forms the highest number of contact edges with protein. Although CARB frequently contacts residues at the β_C_-α_D_ interface, the associated correlation values are generally lower than those observed in the β_A_ and α_B_ contact edges with other glycans. This suggests that allosteric pathways near CARB may be more diverse and potentially more sensitive to local structural fluctuations (SFig. 2, Supplementary Table 2). In contrast, α_B_ and β_A_ form fewer glycan–protein contact edges with α_B_-attached glycan (CARA), but those interactions are more strongly correlated, indicating more consistent and direct communication. Therefore, pathways near the glycan localization site in β_C_ and α_D_ exhibit greater disruption in allosteric coupling compared to β_A_ and α_B_. The introduction of the third glycan diversifies subunit connectivity around the glycan site, causing subunit displacement and altering communication pathways more prominently in β_C_ and α_D_.

### Three α subunits lead to substantial rearrangements and closure of TMD in βα^G^βα^G^α^G^

**Figure 8:**
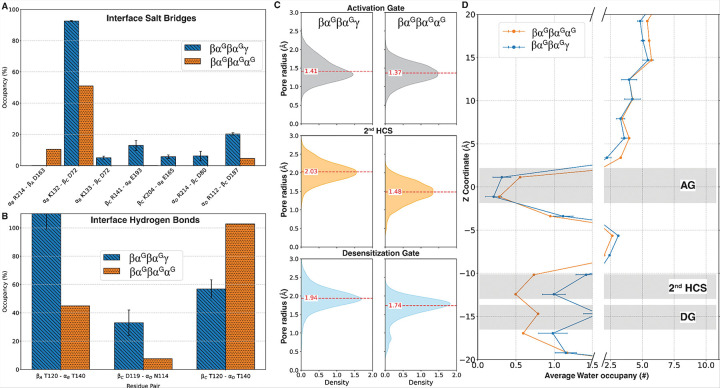
Effect of additional α subunit with three pore-facing glycosylations in βα^G^βα^G^α^G^. A) Changes in salt bridges at the native interfaces. B) changes in hydrogen bonds at native interfaces. C) pore radius comparison at AG, 2^nd^ HCS, and DG. D) Reduction in water occupancy in lower parts of TMD in the βα^G^βα^G^α^G^ system compared with βα^G^βα^G^γ.

The βα^G^βα^G^α^G^ system with three pore-facing glycans exhibited a similar disruption of ECD subunit interface and substantial rearrangements within the TMD as βα^G^βα^G^γ*^G^, ultimately leading to closure in the lower regions of the TMD that renders the channel non-functional. By the end of the simulation, the modeled α_D_-α_E_ interface retained over 90% similarity to previously reported α-homomeric interfaces (PDB ID: 8BHQ), supporting the validity of the system’s construction. In contrast, other subunit interfaces showed a significant reduction in the occupancy for previously identified salt bridges (e.g., α_B_ K132 - β_C_ D72) and even a complete loss of other contacts (Fig. 8A). Similarly, hydrogen bond analysis showed a drastic rearrangement at the β_A_ - α_B_ and β_C_ - α_D_ interfaces (Fig. 8B). Similar to βα^G^βα^G^γ*^G^, the glycan localization at the β_C_ - α_D_ interface in βα^G^βα^G^α^G^ and the steric clash of glycans likely contributes to the observed structural effects and the subtle yet notable 21.66 Å^2^ increase in convex volume of the ECD (SFig. 5) compared to βα^G^βα^G^γ.

The TMD of βα^G^βα^G^α^G^ undergoes significant rearrangement within the first 200 ns of simulation (SFig. 5F). This rearrangement is characterized by a 2.61 Å inward displacement of α_E_ TM2, disrupting its five-fold symmetry of TMD (SFig. 5G). This shift leads to a substantial reduction in pore radius at the 2nd HCS, where the pore radius decreased from 2.03 Å to 1.48 Å (p-value < 0.001), and the desensitization gate goes from 1.94 Å to 1.74 Å (Fig. 8C, SFig. 5). In contrast, the change in pore radius at AG is negligible. This narrowing in the lower pore region corresponds with a marked reduction in water occupancy within the lower TMD (Fig. 8D). By comparison, a structurally similar pentamer without glycosylation (βαβαα) maintained significantly larger pore radii across the three TMD regions of interest (AG: 2.31 Å, 2nd HCS: 2.19 Å, DG: 1.87 Å). These findings indicate that the presence of three pore-facing glycans in the βα^G^βα^G^α^G^ system produced substantial structural perturbations in both the ECD and TMD, showing significant disruption to channel architecture that likely renders the channel non-functional. This is consistent with our observations in the βα^G^βα^G^γ*^G^ system, which also contains three pore-facing glycans.

### Two adjacent α subunits facilitate channel closure in βα^G^α^G^βγ

**Figure 9:**
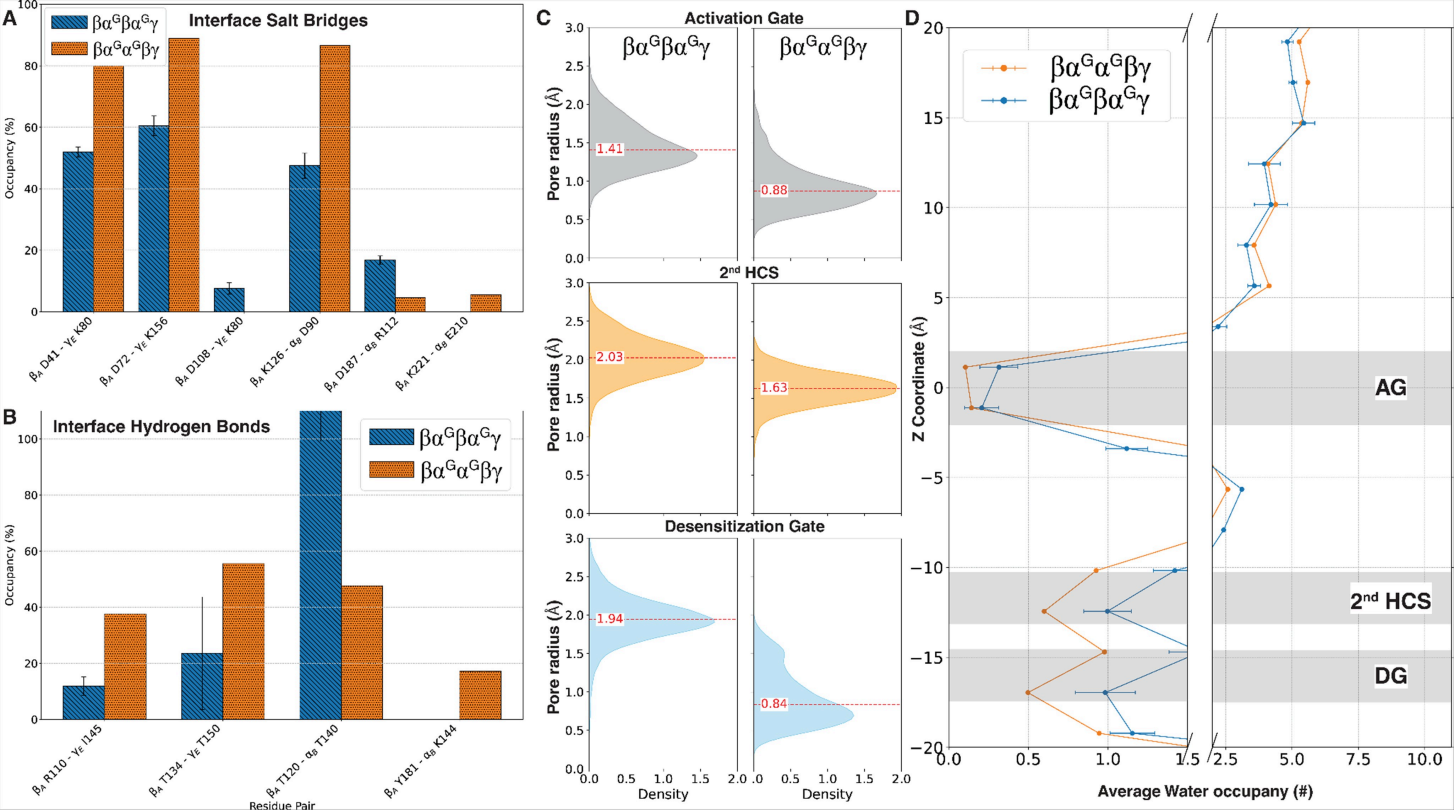
Effect of glycosylation on adjacent α subunits in βα^G^α^G^βγ. A) Changes in salt bridges at the native interfaces. B) changes in hydrogen bonds at native interfaces. C) pore radius comparison at AG, 2^nd^ HCS, and DG. D) Reduction in water occupancy in lower parts of TMD in the βα^G^βα^G^α^G^ system compared with βα^G^βα^G^γ.

The disruption of natural interfaces and steric clashes between glycans in βα^G^α^G^βγ propagate to the TMD, leading to significant closure at the AG and DG. The modeled α_B_ - α_C_ and β_D_-γ_E_ interfaces retained over 90% similarity to previously reported structures (PDB ID: 7QNA, 8BHQ) by the end of the simulation, confirming the validity of the system’s construction. The adjacency of the glycan creates a steric clash at the glycan core, increasing the overall structural fluctuation of glycan and the internal loops they are attached to (SFig. 3). At the ECD, salt bridge occupancy mostly increases at the β_A_-α_B_ and γ_E_-β_A_ interfaces (Fig. 9A). Hydrogen bond analysis follows a similar trend of increase in occupancy. However, the salt bridge β_A_ D187 - α_B_ R112 and the conserved hydrogen bond β_A_ T120 - α_B_ T140—located close to the GABA binding site—showing a notable decrease in occupancy (Fig. 9A&B).

These structural rearrangements at the ECD propagate to the TMD, culminating in a 5.86 Å inward movement of α_C_ and 5.95 Å outward movement of α_B_ at the desensitization gate (SFig. 6). This shift leads to significant tightening at the AG, 2nd HCS, and DG (all p-values < 0.001), which becomes more pronounced as the simulation progresses (Fig. 9, SFig. 6E). Interestingly, a similar reduction in pore radius is not observed in a βααβγ system without glycosylation (SFig. 6F). Water occupancy analysis in βα^G^α^G^βγ further reflects this trend, with a marked reduction in water molecules in the lower TMD similar to βα^G^βα^G^α^G^ (Fig. 9D). Therefore, the rearrangement of the ECD interface drives the progressive closure of both the activation and desensitization gates in βα^G^α^G^βγ. The cumulative effects at both the ECD and TMD indicate a considerable disruption in channel activity for βα^G^α^G^βγ, which would likely render the channel non-functional.

## DISCUSSION

Our study suggested that the pore-facing N-linked glycan on GABA_A_R α subunits plays a crucial role in proper pentameric assembly and channel gating. MD simulations reveal that the introduction of additional or neighboring glycans disrupts native salt bridges and hydrogen bond networks at key β^+^/α^−^ subunit interfaces. This disruption translates into permanent closure of the TMD at the activation or desensitization gates. This observation is not only in line with the previous structural hypothesis that steric hindrance from pore-facing glycans prevents the formation of a pentameric receptor with more than 2 α subunits^37^, but also provide a mechanistic explanation as to how additional or neighboring α subunits alter channel structure and function.

To date, direct experimental evidence supporting the determinant role of pore-facing glycans in GABA_A_Rs assembly pattern remains limited. However, the importance of the α subunit in subunit arrangement is supported by experimental studies suggesting two clusters of residues on the α subunit to be essential for recruiting and stabilizing the interface with the β subunit ^32^. These clusters include the majority of the rearranged salt bridges identified in our simulations, indicating that the additional glycan, by destabilizing native inter-subunit interactions, may impair subunit recruitment or channel activity. Earlier homology modeling studies proposed a symmetrical salt bridge network at the subunit interface^98^, hypothesized to be critical for structural and functional stability. Although more recent structural data shows that several of these proposed salt bridges are spatially distant^45,96,99^, our analysis reveals an alternative salt bridge network that plays an equivalent structural role. This network is primarily located at the lower part of the ECD and extends across the ECD–TMD interface and associated loops (Fig. 4E).

Many disrupted salt bridges and hydrogen bonded residues represent critical nodes for channel function and their mutations are associated with diseases. Notably, the β K126 - α D90, α K132 - β D72, and γ K156 - β D72 interactions form a conserved framework at the β+/α−, α+/β−, and β+/γ− interfaces (Fig. 4)). The residue D90 in the α subunit emerges as a key node, forming salt bridges with β subunits. Pathogenic mutations at this site—such as D90N and D90Y—have been linked to Juvenile Myoclonic Epilepsy and Idiopathic Generalized Epilepsy^100^, underscoring its essential role in subunit coupling and gating. Likewise, the D72Y in the β subunit plays a hub role in this intersubunit salt-bridge network. Its mutation is primarily associated with prostate cancer^100^, indicating that this residue may have broader functional significance. Additionally, mutations such as R112Q^101^, H137T^100^, and D125N^102^ in the α subunit, as well as D119H^100^ and N327K^102^ in the β subunit, are reported in various forms of epilepsy, cancer, and intellectual disability. Importantly, α K306 (SFig. 1), located along a suggested allosteric pathway^103^ for pLGIC, shows disease relevance through the K306T mutation that is implicated in developmental and epileptic encephalopathy^101^. Collectively, the overlap between intersubunit interaction networks and disease-associated mutations highlights the critical structural role of these bonds and their potential vulnerability to glycosylation-induced structural rearrangement.

Previous investigations on GLIC gating identified D32 as a critical molecular “switch” at the ECD–TMD interface^103^. The breaking of the D32–R192 salt bridge is among the initial events triggering structural rearrangements that lead to pore collapse and channel closure. Once freed from R192, D32 engages K248, directly linking ECD rearrangements to TMD movements critical for gating. Similarly, in our study we observed consistent disruption of the homologous salt bridge between α_B_ E76 (equivalent to D32) and β_C_ K306—with occupancy reduced by over 10% across all replicas. Dynamical network analysis also revealed rerouting of allosteric pathways, effectively bypassing the E76 hub, pushing the system toward novel interactions that lead to pronounced TMD closure. Together, these results confirm the conserved, critical role of this salt-bridge interaction in coupling extracellular conformational changes to channel gating in pLGIC.

Comparative analysis of the A–E (β4–β5) linker dynamics reveals that the loop’s deviation from its optimal flexibility—either increased or decreased—can impair channel gating. Previous work by Venkatachalan and Czajkowski ^104^ showed that increasing the flexibility of this loop through the insertion of glycines disrupted gating, with modifications in the β subunit producing a substantial increase in GABA EC_50_ and slower channel opening compared to equivalent insertions in the α-subunit. However, other structural alterations were not examined. Our simulations show that additional pore-facing *N*-linked glycans reduce A–E loop flexibility, particularly in the β_C_ and γ_E_ subunits, which correlates with activation-gate closure in the TMD. Together, these findings indicate that both excessive flexibility and increased rigidity of the A–E loop can disrupt its role in coupling agonist binding to channel opening, defining a narrow dynamic range essential for efficient gating.

Our simulations reveal that pore-facing glycans exert a significant influence on the structural integrity of both the activation gate (9′), the desensitization gate (− 2′) and 2nd HCS (2′) within the TMD of the GABA_A_R. In the βα^G^βα^G^γ, the average pore radii at 9′, 2′, and − 2′ positions were 1.38 Å, 2.03 Å, and 1.96 Å, respectively—values consistent with a semi-desensitized or pre-active state, yet notably narrower than the radii suggested in open-state models, where the 9′ gate reaches ~ 4 Å and the − 2′ gate opens to ~ 3 Å in a computational study^105^. βα^G^βα^G^γ*^G^ with three pore-facing glycans exhibited a pronounced constriction at the 9′ gate (1.19 Å) and moderate reductions at the 2′ (1.86 Å) and − 2′ (2.05 Å) positions in addition to exhibiting a rapid switching behavior of the AG at 9′ between 0.6 Å and 1.25 Å. This is indicative of a closed-like state. This narrowing becomes more severe in βα^G^βα^G^α^G^, particularly at 2′ (1.48 Å) and − 2′ (1.74 Å), with the 9′ gate measuring 1.37 Å. The βα^G^α^G^βγ showed the most constricted profile, with pore radii falling to 0.88 Å (9′), 1.63 Å (2′), and 0.84 Å (− 2′), values well below the thresholds required for chloride conduction. Compared to literature-reported states stabilized by modulators such as etomidate or propofol—where AG opens widely (4.2–5.2 Å) but DG remains collapsed (~ 1.4–1.6 Å)^34,44^—our glycan-containing mutants consistently show closure at both gates, suggesting a shift toward a non-conductive conformation. Notably, channel closure does not correlate with GABA dissociation, as it does not necessarily occur before or after GABA dissociation, indicating that the observed constrictions at AG, 2nd HCS, and DG are not a consequence of GABA dissociation.

The glycosylation site is conserved across all GABA_A_R α subunits and may represent a recent evolutionary gain-of-glycosylation event. This site is unique to α subunits, as this sequon is absent in other GABA_A_R subunits or other members of the pLGIC family. Notably, the functional importance of this region extends beyond the conserved *N*-glycosylation site. For instance, in the β subunit, the homologous extracellular β4–β5 loop—although not glycosylated—plays a key role in receptor activation^104^. Furthermore, the evolutionary conservation of a threonine residue (Fig. 5G) located two positions downstream of the glycosylation site highlights its role as a structural “priming” element that facilitates the emergence of glycosylation sites^106^. Glycosylated asparagine residues are subject to strong purifying selection pressure and thus evolve more slowly^107^. However, sequence analyses have shown that most newly acquired *N*-glycosylation sites arise from the introduction of asparagine into pre-existing motifs already containing a conserved threonine at the + 2 position (Fig. 2B&C)^106,108^. These latent motifs, maintained by genetic drift or bias, serve as evolutionary placeholders—structurally stable regions poised for rapid functional enhancement upon acquiring a glycosylation-competent sequon^109^. A well-documented example of this mechanism is found in primate thyroglobulin evolution, where a threonine residue conserved across mammals was complemented by a human-specific asparagine mutation at position 76, generating an *N*-glycosylation site (*N*-X-T) that significantly improved thyroxine production^106^. Similarly, in GABA_A_Rs, conserved threonine residues may act as evolutionary precursors for glycosylation, contributing to structural integrity while enabling future regulation of channel assembly and function.

While our computational findings implicate the determinant role of pore-facing glycans in subunit composition to prevent three or neighboringα subunits, experimental validation of this mechanism remains underexplored and challenging. Previous structural studies that resolved GABA_A_R α homomers involved substantial modification to the ECD, particularly at the pore-facing N-glycosylation site. For example, one study employed a cell line with incomplete glycan expression, combined with a neutralizing mutation at N123 on the α1 subunit to prevent glycosylation^47^. Another replaced the GABA_A_R ECD with that of the ELIC’s^48^ or β subunits^110^, thereby eliminating the native pore-facing glycosylation. Several studies have proposed an α–α interface using concatenated constructs designed to enforce specific subunit arrangement^26^. However, these findings remain controversial, as no naturally resolved structure has confirmed such configurations, and it is argued that forced assembly may yield non-native, potentially deleterious receptor builds^111^. A more rigorous approach to validate the role of glycosylation in subunit arrangement would be to resolve the structure of a fully concatenated construct encoding all five subunits, accompanied by detailed glyco-profiling using mass spectrometry.

It is noted that there are several limitations to the current study. First, the structure of the pore-facing glycan modeled in our simulations may not fully reflect its native composition. Although, current cryo-EM structures show that the pore-facing glycan on the α subunit adopts a high-mannose form^21,25,37,44^, with one structure displaying a Man8 configuration^44^, additional studies are needed to clarify the extent of terminal mannose trimming. This uncertainty stems from the inherent variability in glycosylation processing by enzymes in the endoplasmic reticulum and *cis*-Golgi ^112,113^. If the pore-facing glycans are more extended *in vivo*, their structural and functional impact could be greater than what we observe in our present study. Furthermore, N-linked glycosylation composition can vary across tissues and species^112^. Future in-depth glycomic profiling of GABA_A_Rs may reveal additional insights into their regulation by glycosylation. Secondly, this study specifically examines the effects of pore-facing glycosylation on the structure and dynamics of the assembled GABA_A_Rs. However, we do not address its potential role in the assembly process. Given that these N-linked glycans are known to influence protein expression, assembly, and trafficking^46^; this remains an important yet unexplored aspect beyond the scope of our simulation study. Further studies are needed to investigate how glycosylation affects receptor biosynthesis and maturation.

## CONCLUSION

Our study reveals that pore-facing N-linked glycans on GABA_A_ receptor α subunits play a critical role in determining pentameric assembly patterns and modulating channel gating. Through integrative structural survey, sequence analysis, and molecular dynamics simulations, we demonstrate that introducing two neighboring pore-facing glycans disrupts conserved interfacial networks—specifically salt bridges and hydrogen bonds—at key β+/α− subunit interfaces within the ECD. These disruptions propagate allosterically from the ECD to the TMD, leading to altered internal loop flexibility, loss of coordinated gating motions, and premature closure of the activation or desensitization gates. This structural rearrangement results in a shift toward non-conductive channel conformations. These computational insights are consistent with prior structural hypotheses and help explain functional consequences of disease-associated mutations located near glycan-sensitive regions or along the ECD-TMD allosteric coupling pathway. Importantly, our findings underscore the evolutionary uniqueness and functional importance of the α subunit-attached pore-facing *N*-glycosylation site, suggesting it acts as a molecular determinant of subunit composition and channel function. Although our models provide mechanistic clarity, experimental validation remains essential. Together, our findings reveal the underappreciated yet critical role of pore-facing glycans in shaping GABA_A_R architecture and function. This work lays the foundation for glycan-aware strategies in receptor modulation and opens new avenues for developing selective therapeutics targeting glycosylation-mediated control of ion channel activity.

## Supplementary Material

Supplementary Files

This is a list of supplementary files associated with this preprint. Click to download.

• SupplementaryMaterials.docx
